# Do Students with Varying Academic Ability Benefit Equally from Personal Qualities? Applying a Trait and State Perspective

**DOI:** 10.1007/s11162-018-9498-y

**Published:** 2018-02-26

**Authors:** A. Kool, M. T. Mainhard, A. D. C. Jaarsma, P. van Beukelen, M. Brekelmans

**Affiliations:** 1 Faculty of Social and Behavioural Sciences, Utrecht University, P.O. Box 80140, 3508 TC Utrecht, The Netherlands; 2 Faculty of Veterinary Medicine, Utrecht University, Utrecht, Netherlands; 3 Center for Research and Innovation in Medical Education, University Medical Center Groningen, Groningen, Netherlands

**Keywords:** Perseverance, Intellectual curiosity, Academic achievement, Academic ability, Moderation

## Abstract

Using multilevel models, this study examined whether students with varying academic ability benefit equally from perseverance and intellectual curiosity in terms of academic achievement. In addressing this question two perspectives were applied: a trait perspective, focusing on differences between students, and a state perspective, focusing on differences within students across semesters. By means of an online questionnaire, undergraduate students (N = 2272) were asked to rate themselves on perseverance and intellectual curiosity at the beginning of five consecutive semesters. Results indicate that academic ability but also personal qualities have to be taken into account to explain the differences between students in academic achievement. In particular perseverance was found to be important in explaining differences both between students and within students across semesters. Also, individual students fluctuate quite substantially in their reported perseverance and intellectual curiosity from semester to semester.

## Introduction

This study examines whether associations between personal qualities of students and their academic achievement depend on students’ general academic ability (Heaven and Ciarrochi 2012; Ziegler et al. 2009). With the greater emphasis on students’ academic achievement in higher education, student achievement or a student’s grade point average (GPA) is a vital quantifier (Brown and Campion 1994; Thoms et al. 1999). Consequently, substantial effort has been made to understand why certain students are more successful at achieving high grades than others (e.g., Chamorro-Premuzic and Furnham 2008; Poropat 2009). Traditionally, ‘being smart’ (i.e., a student’s general academic ability) has been considered the most significant predictor of students’ academic achievement (Kuncel et al. 2004). Over recent decades, however, there has been growing interest in the role of factors other than general academic ability or intelligence in predicting grades (Chamorro-Premuzic and Furnham 2008; Duckworth and Yeager 2015; Richardson et al. 2012; Rosen et al. 2010; von Stumm et al. 2011). One group of variables that has generated a considerable amount of attention for predicting academic achievement is student personality (i.e., *conscientiousness, extraversion, neuroticism, openness, and agreeableness*; O’Connor and Paunonen 2007; Richardson et al. 2012). Studies that have related personality to academic achievement have shown that, specifically, aspects related to conscientiousness (i.e., being diligent, hardworking, and dutiful) and, to a lesser extent, openness (i.e., being creative, intellectual, and curious) predict academic achievement to a greater extent than general academic ability or intelligence (Noftle and Robins 2007; Poropat 2014).

Although the importance of those personal qualities for academic achievement has been confirmed, the degree to which the qualities are related to academic achievement varies considerably between studies (O’Connor and Paunonen 2007; Poropat 2009). Consequently, several studies have pointed to the presence of possible moderating variables that affect the relation between personal qualities and academic achievement (Poropat 2014; Ziegler et al. 2009). The present study examines to what extent a student’s academic ability influences the relation between academic achievement and two aspects of conscientiousness and openness that are most relevant to, and most often used in, the academic context: (1) *perseverance* overlaps with the achievements facets of conscientiousness, but has greater emphasis on long-term goals rather than on short-term intensity, which is important within educational contexts (Duckworth et al. 2007) and (2) *intellectual curiosity* is related to openness and this facet of openness is most commonly associated with learning and academic achievement (Poropat 2014).

### Academic Ability as a Moderator

In the literature, there are many constructs that refer to a student’s potential to perform well in an academic context, such as academic ability, cognitive ability, and intelligence. Depending on the operationalization of these constructs, the different terms often overlap or are used interchangeably. Nevertheless, previous studies have shown that one’s potential to perform well in the academic context may play a complex role in the personality-achievement relationship. For example, Heaven and Ciarrochi (2012) showed that openness was positively associated with academic achievement among students with high cognitive ability scores but not among students with lower scores. Beaujean et al. (2011), however, found that openness had a stronger positive effect on mathematics scores among students with lower intelligence compared to students with high intelligence. Ziegler et al. (2009) studied the interaction between intelligence and achievement striving (a facet of conscientiousness) in predicting academic achievement. After splitting the sample into high-performing and low-performing student groups, achievement striving had an enhancing effect on academic achievement in low performers, while the opposite effect was found for high performers.

Academic ability has also been used as a moderator in studies examining the relation between academic achievement and other personality variables. Lozano et al. (2014), for example, investigated the role of ability in the relation between impulsivity and academic achievement. Among students high in ability, high levels of impulsivity were associated with lower academic achievement. Among students with lower ability scores, this relation between impulsivity and academic achievement was almost non-existent. Overall, these studies indicate that academic ability may play an important role in explaining why not all students may benefit equally from personal qualities.

### Trait and State Perspectives

To date, studies investigating the effects of personal qualities on academic achievement have typically aimed to explain why certain students are more successful than others using average levels of achievement (GPA) and measures of personal qualities at one moment in time (Cheng et al. 2012). This approach implicitly assumes that personal qualities are stable, trait-like characteristics that are similar across courses and teachers and are not responsive to experiences or educational programs (Duckworth and Yeager 2015). Since previous studies have shown relative stability of the Big Five dimensions (Richardson et al. 2012), it indeed seems likely that there are individual differences in general levels of personal qualities.

However, Specht et al. (2011) showed that the Big Five constructs changed not only due to maturation but also in reaction to the experience of life events. Moreover, individuals show variations in their behavior in reaction to situational changes (Fleeson and Wilt 2010). This suggestion of more state-like variability in personal qualities is in line with aims of educational programs, especially for young adults.

In this study, we therefore not only examine how academic ability affects the relation of perseverance and intellectual curiosity with academic achievement *between students* (trait perspective) but also extend the present literature by exploring this moderation mechanism *within students* (state perspective). That is, we also focus on individual students’ fluctuations in academic achievement and their personal qualities.

### The Current Study

In the current study, we examined how academic ability affects the associations of perseverance and intellectual curiosity with academic achievement and specifically explored whether students with different academic abilities benefit equally from personal qualities. Given the relatively sparse body of literature on this topic and contradicting findings from earlier studies, we generated no specific hypotheses concerning the direction of the moderation effects.

Moreover, we applied a trait-like perspective focusing on differences *between* students regarding the relationship of perseverance and intellectual curiosity with academic achievement. Additionally, the state perspective represented a more situational point of view and focused on variability within students across semesters.

## Method

### Participants and Procedure

Undergraduate students at a large research university in the Netherlands participated in the present study, which was part of a larger research project on academic achievement in undergraduate programs. Students in 17 different undergraduate programs (i.e., Science, Humanities, Medical Sciences, Social Sciences, Geosciences, Law and Economics, and Liberal Arts and Sciences) were invited to report on their perseverance and intellectual curiosity by means of an online questionnaire. Data on academic ability and academic achievement were collected from university files.

Students were invited to participate by an email stating the goal of the study, and upon invitation, students had 2 weeks to decide whether to participate. It was made clear that all information would be treated as strictly confidential and would only be used for scientific research. After opening the link to the online questionnaire provided in the email, students were first asked to provide their consent to participate and for access to their university files to retrieve information on achievement and pre-university final exam grades.

For each semester between February 2010 and September 2012 (i.e., five-wave repeated-measures design), we gathered data on students in their first to fourth years (i.e., eight semesters) of their undergraduate program. In total, 23,276 questionnaires were completed by 11,392 individual students (66% female; response rate 38%). To study variations between students (trait perspective) and within students (state perspective), we selected students with data available for at least three waves. This resulted in a sub-sample of 2272 students (73% female; 3 waves: 43%, 4 waves: 40%, 5 waves: 17%; N = 8516 questionnaires).

### Measures

#### Perseverance

Perseverance overlaps with the achievement aspects of conscientiousness but has a greater emphasis on long-term goals than short-term intensity (Duckworth et al. 2007). We used three items from the Perseverance of Effort subscale from the Grit questionnaire by Duckworth and Quinn (2009). Students were asked to indicate the degree to which the following items were an adequate description of themselves: ‘I finish whatever I begin’; ‘I am diligent’; and ‘I am a hard worker’. Items were assessed on a Likert scale ranging from 1 (not at all true of me) to 7 (very true of me). The reliability (Cronbach’s α) was 0.77. Data were gathered at the start of the semester. *Student*-*averaged* scores (based on at least three measurements) represented general, trait-like perseverance.

#### Intellectual Curiosity

Intellectual curiosity was measured with the items reflecting the facet of the Openness to Experiences scale (Gerris et al. 1998; Goldberg 1992). Students were asked to indicate the degree to which an adjective was an adequate description of themselves (seven-point Likert scale ranging from 1 (disagree) to 7 (agree)). The subscale consisted of three items (α = 0.60): ‘I am innovative’; ‘I am inventive’; and ‘I am inquisitive’. Data were gathered at the start of the semester. *Student*-*averaged* scores (based on at least three measurements) represented general, trait-like *intellectual curiosity.*

#### Academic Achievement

A semester GPA score was computed as a measure of academic achievement during the entire semester. In the Netherlands, grades range from 1 (lowest) to 10 (highest). To pass an exam, a score of at least 5.5 is required for each course; therefore, the semester GPA of participants range between 5.5 and 10. At the university where the present study was conducted, semesters were generally composed of four courses (representing 210 h of study time each).

#### Academic Ability

Academic ability is often measured by standardized tests, such as the Scholastic Assessment Test (SAT) in the United States (Noftle and Robins 2007). In the Netherlands, no SAT scores are available. However, all pupils receiving high school, pre-university education take the same final exams for their school subjects. Therefore, we used students’ GPA scores from their pre-university education exams as an indicator of their academic ability. Exam GPA scores were drawn from the university’s files.

### Analyses

We used a repeated-measures design and applied both a trait and state perspective. We performed a multilevel analysis (SPSS MIXED, version 24) with GPA as the dependent variable and semesters as the first-level, repeated-measures units. *Students represented the second*-*level units.* We used panel modeling (Hox 2011) because we did not expect GPA to show a pattern of growth or decline across the semesters of the undergraduate program (i.e., unstructured change modeling).

Level 1 predictors (i.e., time variant predictors: state-like perseverance and intellectual curiosity) were student-centered. Level 2 predictors (trait-like perseverance and intellectual curiosity) were grand mean-centered. This approach allowed us to differentiate between (a) trait-like or between-student effects of general levels of perseverance and intellectual curiosity and (b) state-like or within-student effects of fluctuations across semesters in perseverance and intellectual curiosity.

In the unconditional Model 0 (M0), we decomposed the variance in GPA into between-student and within-student (across semester) variance.

In Model 1 (M1), we tested the effects of trait-like perseverance and intellectual curiosity on GPA at the between-student level. Information on the role of academic ability is provided by M1b.

In Model 2 (M2), we tested the effects of the time variant predictors at the within-student level. To examine whether associations between state-like perseverance and intellectual curiosity with GPA varied between students, random slope models were tested on a variable-by-variable basis, as suggested by Hox and colleagues (2010). Only random slopes that showed a significant improvement in the model fit are presented, and only those variables’ cross-level interactions with academic ability were examined.

Full maximum likelihood estimation was used when both regression coefficients and variance components were tested, and restricted maximum likelihood estimation was used when variance components were involved (Hox et al. 2010). For the model comparison, we used deviance tests. Given that the explained variance (at level 1 or 2) is the analog of R2 change in the OLS regression, we adopted Cohen’s (1988) guidelines for effect sizes, i.e., 0.02, 0.13 and 0.26, which represented a small, medium, and large effect, respectively.

#### Assumptions

Level 1 and level 2 standardized residuals in both the intercept-only and final model were inspected (Hox et al. 2010). To test for outliers, we assessed the number of standardized residuals greater than 3.29 (Tabachnick and Fidell 2007, p. 73). An inspection of (detrended) normal Q–Q plots suggested normal distributions of all residuals (skewness, kurtosis between 0.17 and 0.73). An inspection of the plots of standardized residuals against predicted values and bivariate scatterplots did not indicate strong violations of assumptions of linearity or homoscedasticity. In a single-level regression analysis, collinearity diagnostics provided no cause for concern (condition indices < 2, Tabachnick and Fidell 2007, p. 91). In view of the large N, we concluded that the outliers were within an acceptable range (below 0.30% in Model 0, below 0.5% in the final model). Since testing the final model with and without outliers revealed comparable conclusions, the results are presented with outliers included.

## Results

The descriptive statistics for perseverance, intellectual curiosity, academic achievement, and academic ability are displayed in Table 1 at the student level.

**Table 1:** Descriptive statistics of the study variables (student level, N = 2272)

	1	2	3	M	SD	Min	Max
1. Academic achievement	–			7.11	0.55	6.00	9.29
2. Academic ability	0.59**	–		7.18	0.65	5.50	9.23
3. Perseverance	0.35**	0.21**		5.29	0.88	1.67	7.00
4. Intellectual curiosity	0.07**	0.11**	0.24**	4.96	0.70	2.44	7.00

**p < 0.01

Results of the multilevel analyses are presented in Table 2. The intercept-only model showed significant random variance in GPA with a substantial amount of variance (40%) within students (intraclass correlation coefficient ICC = 0.60), demonstrating the relevance of both the trait and state perspectives when explaining differences in GPA. For perseverance and intellectual curiosity, the ICCs were 0.69 and 0.64, respectively.

**Table 2:** Academic achievement explained by inter- and intra-individual differences in perseverance, intellectual curiosity, and academic ability

		Inter-individual trait perspective				Intra-individual state perspective	
	M0	M1a		M1b		M2	
	B (SE)	B (SE)	B	B (SE)	β	B (SE)	β
Fixed part							
Intercept	7.11 (0.01)	7.11 (0.01)		7.10 (0.01)		7.10 (0.01)	
Level 2 effects							
Perseverance-trait (P-t)		0.22 (0.01)**	0.19	0.16 (0.01)**	0.14	0.16 (0.01)**	0.14
Intellectual curiosity-trait (I-t)		− 0.02 (0.02)	− 0.01	− 0.04 (0.01)**	− 0.03	− 0.04 (0.01)**	− 0.03
Academic ability (AA)				0.45 (0.01)**	0.29	0.45 (0.01)**	0.29
AA*P-t				0.10 (0.02)**	0.06	0.10 (0.02)**	0.06
AA*I-t				− 0.04 (0.02)*	− 0.02	− 0.05 (0.02)*	− 0.02
Level 1 effects							
Perseverance-state (P-s)						0.08 (0.01)**	0.04
Intellectual curiosity-state (I-s)						0.02 (0.01)*	0.01
Random part							
Level 1 residual variance	0.174 (0.003)**	0.174 (0.003)**		0.174 (0.003)**		0.167 (0.003)**	
Level 2 intercept variance	0.255 (0.009)**	0.218 (0.008)**		0.130 (0.005)**		0.132 ( 0.005)**	
Intercept-slope covariance P-s						0.015 (0.004)**	
Random slope P-s						0.015 (0.005)**	
−2 Loglikelihood	13481.26	13183.54		12270.62		12167.344	
Δ − 2 Loglikelihood		297.72** (vs. M0)		912.92** (vs. M1a)		103.28** (vs. M1b)	

*Note* no significant interactions between perseverance and intellectual curiosity

*p < 0.05, **p < 0.01

To explain inter- and intra-individual differences in GPA, we tested different multilevel models. Note that the large sample size of the current study increased chances of finding statistically significant effects, therefore the effect sizes should be considered for a better understanding of the substantive importance of the predictors.

In Model 1a, we included trait-like personal qualities to explain the differences between students (i.e., inter-individual perspective). The model improved significantly compared to the intercept-only model; χ^2^ (2) = 297.72, *p* < 0.01. Trait-like perseverance explained 14.5% of the variance at the between-student level (medium effect size, Cohen 1988). When academic ability was also included to explain differences between students (model M1b), we found a significant main effect and a significant moderation effect for both perseverance and intellectual curiosity. The model improved significantly compared to the model with only personal qualities (χ^2^ (3) = 912.92, *p* < 0.01) and explained 48.9% of the inter-individual differences in students’ GPA scores (large effect size, Cohen 1988). For perseverance, there was a positive main effect and a positive interaction effect with academic ability. Perseverance strengthened achievement and this effect was stronger for students with higher ability. These students benefited more from perseverance than students with lower ability who had similar reported levels of perseverance. Unexpectedly, for intellectual curiosity, there was a negative main effect and a negative interaction effect with academic ability. Intellectual curiosity weakened achievement, and this effect was stronger for students with higher ability (controlling for the effect of perseverance). However, the specific effect size of the interaction of academic ability and personal qualities was small in terms of explained variance (1.2%).

In Model 2, we tested the effects within students (i.e., intra-individual perspective). For perseverance, the effect was in the same direction as the between-student effect: reporting higher perseverance at the beginning of a semester was on average associated with higher achievement during that semester. This effect differed across students (i.e., significant random slopes, 95% CI [−0.16; 0.32]). On average and within individual students, each point increase on the perseverance scale resulted in an increase in academic achievement of 0.08 points during that semester (i.e., 0.15 *SD*, small effect size, Cohen 1988). There was no additional moderating mechanism (cross-level interaction) of academic ability; *B* (*SE*) = 0.02(0.02), *p* = 0.256; χ^2^ (1) = 1.29, *p* > 0.05. A significant intercept-slope covariance indicated that better students (in terms of GPA) benefitted more from extra perseverance in a given semester (*r* = 0.35). In contrast to the between-student effect of intellectual curiosity, the within-student effect was positive: relatively higher intellectual curiosity during a semester was associated with somewhat higher grades during that semester. This effect was small and did not vary across students (i.e., no significant intercept-slope co-variance or random slopes). Together, the predictors added in this step explained only marginal amounts of variance at the within-student level.

For ease of interpretation, we predicted academic achievement scores for students with different combinations of academic ability and perseverance (based on Model 2, Table 2). We compared the means of the predicted scores for academic achievement of three groups of students: 10% of the students with the lowest academic ability scores (5.5–6.4), 10% of the students with scores around the median for academic ability (7.0–7.2), and 10% of the students with the highest scores (8.1-9.2). Within each ability group, we predicted scores for the 10% highest perseverance scores, 10% around the median, and 10% lowest scores (see Table 3). The predicted scores are in line with observed sample estimates.

**Table 3:** Prediction of mean GPA for students with different levels of academic ability and perseverance (10% high, median, and low)

			Perseverance		
			High	Median	Low
			6.3–7.0	5.3–5.5	1.7–4.1
Academic ability	High	8.1–9.2	8.11	7.69	7.16
	Median	7.0–7.2	7.22	6.95	6.86
	Low	5.5–6.4	6.81	6.77	6.63

According to the predictions from the multilevel model presented in Table 3, students with low academic ability benefited less from perseverance (predicted difference in GPA = 0.18 between low and high perseverance, i.e., 0.27 *SD* in semester GPA, small effect size, Cohen 1988) than students with high academic ability (predicted difference in GPA = 0.95, 1.45 *SD* in GPA, large effect size, Cohen 1988). In the Netherlands, a common admission criterion for honors programs is a GPA of 7.50 or higher. According to the multilevel model, it seemed unlikely that students with low (and even median) academic ability could meet this criterion through extra perseverance alone. Students with high academic ability would need at least average perseverance to meet this criterion. The association of intellectual curiosity with students’ average academic achievement was not as strong or consistent as the effects of perseverance.

To illustrate the combined effect of perseverance and intellectual curiosity, we predicted the scores for students with one standard deviation above and below average academic ability and one standard deviation above and below average perseverance and intellectual curiosity (see Table 4). According to our multilevel model, students with high academic ability and relatively high levels of perseverance but low intellectual curiosity were predicted to be most successful in terms of academic achievement.

**Table 4:** Prediction of mean GPA for students with different academic ability, perseverance and intellectual curiosity (± 1 SD)

		Perseverance/intellectual curiosity			
		+ 1 SD/+ 1 SD	+ 1 SD/− 1 SD	− 1 SD/+ 1 SD	− 1 SD/− 1 SD
Academic ability	+ 1 SD	7.72	7.90	7.20	7.38
	− 1 SD	6.72	6.70	6.60	6.58

## Discussion

In the current study, we investigated how academic ability affects the association between personal qualities and academic achievement and specifically whether students with varying academic ability benefit equally from personal qualities. By using multilevel panel modeling for repeated measures, we were able to examine the effects of academic ability on the relationship of perseverance and intellectual curiosity with academic achievement from both a trait-like perspective, focusing on differences between students, and a state-like perspective, focusing on differences within students across semesters.

Based on the results, the main conclusion is: academic ability but also personal qualities have to be taken into account to explain the differences between students in academic achievement. In the current study, perseverance and intellectual curiosity were included as personal qualities. For these two qualities, perseverance in particular was found to be important in explaining differences both between students and within students across semesters.

### Inter-Individual Differences–Trait Perspective

From a trait-like perspective, the results showed that the combination of academic ability and perseverance was important. But according to the multilevel model, even a student with median academic ability was very unlikely to meet the grade criterion of an honors program just by working hard. Students with high academic ability were however already likely to meet this criterion with an average perseverance. The association of intellectual curiosity with students’ average academic achievement was not as strong or consistent as the effects of perseverance.

Although all students profited from a generally high level of perseverance during their years of undergraduate study (see also Duckworth and Seligman 2005; Richardson et al. 2012), students with high academic ability benefited slightly more. This finding suggests that it might be worthwhile to take a student’s academic ability into account when studying the relation between personal qualities and academic achievement. This finding is not in line with those of Beaujean et al. (2011), who found that students with lower cognitive ability benefit more from conscientiousness than students with high ability and can somewhat compensate by working hard. These differences may be ascribed to the use of different constructs; for example, Beaujean et al. used math scores to determine achievement.

Unexpectedly, intellectual curiosity only weakly affected students’ performance. The results suggested that intellectual curiosity slightly hampered all students but students with high ability even more so. This finding suggests that in the undergraduate programs that were studied here, being innovative, inventive, and inquisitive was not reflected in higher grades and this was reflected even less in students with higher academic ability. It is possible that students, at least in the current study, were assessed in ways that did not take their intellectual curiosity into account. This seems to conflict with what job recruiters demand, as they often seek innovative and creative employees (Miron et al. 2004). A study by Chamorro-Premuzic (2006) suggests that the relation between intellectual curiosity and grades varies by the method of assessment used to test students. In British university students, scores on openness were positively correlated with grades on final theses, while these scores were more weakly related to block assessments and final exam scores. Indeed, in the university under study, students are mostly assessed by standardized exams. This method of assessment seems to favor students with high perseverance and low intellectual curiosity over others. Given the results of Chamorro-Premuzic (2006), grades on final theses rather than overall GPA may provide job recruiters a better indication of students’ intellectual curiosity, especially in students with high ability. Zhang and Ziegler (2015) further suggest that being innovative may not be beneficial in an academic context for students with high intelligence, since it might lead to distractions and lower interest in the content taught. For students with lower intelligence, this negative effect is possible reduced because the study content may satisfy their higher levels of curiosity.

### Intra-Individual Differences–State Perspective

Since former studies investigating the personal qualities-achievement relationship mainly focused on explaining the differences in achievement between students (Cheng et al. 2012), we also investigated intra-individual fluctuations in achievement. Specifically, it was investigated whether more or less perseverance and intellectual curiosity during a semester had different effects on achievement for students with varying levels of academic ability.

Individual students indeed fluctuated quite substantially in their reported perseverance and intellectual curiosity from semester to semester. Again, the effect of intellectual curiosity was minor, and no moderation effect was found for academic ability. We found, however, that fluctuations in grades were positively associated with fluctuations in students’ perseverance. There was no evidence that this effect was different for students with low versus high academic ability. We found, however, that students who performed better on average at the university benefited somewhat more from extra perseverance in a given semester, that is, a positive association between average ability and the effect of perseverance of *r* = *0*.35 was found. Thus, when studying state-like associations between personal qualities and achievement, general achievement levels seemed to play a role play a role.

### Strengths, Limitations and Future Research

The study was conducted across different educational programs with a rather large sample; in addition, data from Psychology programs were collected more often. Scholars have criticized the overrepresentation of samples based on psychology students in this area of research (Busato et al. 2000; Farsides and Woodfield 2003; Vedel 2014). Although this was not the focus of the current study, we compared students from Psychology programs with students from other programs and indeed found significant main effects of program and interaction effects with personal qualities; however as this was not the primary focus of the current study, we leave further investigations of these associations for future research.

We employed self-reports to measure perseverance and intellectual curiosity. We agree with Duckworth and Yeager (2015) that self-report questionnaires are arguably better suited than any other measure for assessing internal psychological states because they typically ask individuals to integrate numerous observations of thoughts, feelings or behaviors over a specific period of time. The use of self-reports may, however, result in a certain bias. For example, students may respond in a socially desirable or self-serving way (Holden 2007). We tried to mitigate this potential by guaranteeing that the questionnaires would be used for research purposes only and that the results would be presented anonymously. Moreover, future studies using alternative ways to measure personal qualities, such as divergent thinking tests to measure intellectual curiosity (Feist 1998), or the use of other-ratings (such as teachers) instead of self-ratings (Poropat 2014), would be a great addition to the present study.

Furthermore, to gain a relatively large sample, we used high school, pre-university exams as a proxy for academic ability. Scores on the SAT have been reported to be an adequate measure of general intelligence (Frey and Detterman 2004); however, we cannot be certain whether the use of a standardized IQ test would have led to different results.

We recommend that future studies investigate the role of academic ability in the relationship of personal qualities and academic achievement with additional constructs and other possible moderators. This may gain a broader insight into why certain personal qualities may not have the same effect on academic achievement for all students. Also, since the personal qualities in the current study were assessed with measures focusing on rather specific aspects of the qualities, the use of questionnaires that assess a broader spectrum of perseverance and intellectual curiosity may provide further insight on different aspects of the qualities.

On a more general note, our results confirm the assumption of Duckworth and Yeager (2015) that personal qualities change according to the environment and students’ experiences (in our case, from semester to semester) and may indeed be responsive to educational interventions. A next step would be to examine the specific elements of educational courses and programs that may be related to high and low perseverance to delineate the factors that stimulate students’ perseverance.

### Practical Implications

The results of this study yield several practical implications for the practice of higher education. For educators, a first step in supporting students is being aware that stimulating certain qualities may not overcome differences between students due to ability and may also not have the same effect on academic achievement for all students. If future studies confirm our findings, interventions designed to enhance perseverance (e.g., by promoting a student’s belief that abilities can be developed through effort and practice (Dweck 2009)) should maintain reservations regarding the upper bounds of achievement scores that can be attained. Our findings seem to support a more personalized approach when guiding students.

Moreover, fluctuations in perseverance and intellectual curiosity show that personal qualities vary during the academic year. For students and educators, it is important to be aware of this variation. Educators can further guide students in regulating their personal qualities by addressing personal development and reflection in their courses (Korthagen and Vasalos 2005). For educational researchers, the fluctuations in personal qualities indicate that outcomes partly depend on the specific moment that qualities are assessed (generalizability).

Finally, the results suggest that students with high academic ability and perseverance were most successful in our sample, while intellectual curiosity played only a marginal role. Universities should consider whether various methods of assessment and different forms of assignments including essays, oral presentations, or theses may be used to tap into and reward intellectual curiosity among students (Chamorro-Premuzic 2006).
